# Executive functions in agenesis of the corpus callosum: Working memory and sustained attention in the BTBR inbred mouse strain

**DOI:** 10.1002/brb3.1933

**Published:** 2020-12-10

**Authors:** Loren A. Martin, Fang‐Wei Hsu, Brooke Herd, Michael Gregg, Hannah Sample, Jason Kaplan

**Affiliations:** ^1^ Department of Graduate Psychology Azusa Pacific University Azusa CA USA; ^2^ Department of Psychology Azusa Pacific University Azusa CA USA; ^3^ Center for Next‐Gen Precision Diagnostics UCSF San Francisco CA USA; ^4^ U.S. Department of Veterans Affairs Coatesville PA USA

**Keywords:** attention, autism, behavioral paradigms, cognition, RRID:IMSR_JAX:000664, RRID:IMSR_JAX:002282

## Abstract

**Introduction:**

Agenesis of the corpus callosum (AgCC) is characterized by the congenital partial or complete absence of the corpus callosum. Several strains of mice have been reported to carry AgCC, with the BTBR T^+^Itpr3^tf^/J (BTBR) inbred mouse strain consistently showing a complete absence of the corpus callosum, as well as a variable reduction in the size of the hippocampal commissure. While much research has focused on the social deficits of the BTBR strain, little research on its cognitive behavior has been conducted. The goal of our study was to compare two facets of executive functioning, spatial working memory, and sustained attention between the BTBR and C57BL/6J (B6) strains.

**Methods:**

Spatial working memory was measured utilizing a delayed matching‐to‐position (DMTP) task and sustained attention was measured utilizing an operant task in which mice were trained to distinguish signal and nonsignal events.

**Results:**

Both the BTBR and B6 mice demonstrated a predictable decline in performance on the DMTP task as the delay interval increased and predictable increase in performance on the sustained attention task as the duration of the signal event increased. Although no significant differences were found between strains on the performance of these tasks, there was a significant difference in learning the association between lever pressing and food reward. Histological investigation confirmed the complete absence of commissural fibers from the corpus callosum, but also the hippocampal commissure, counter to a previous study.

**Conclusion:**

The results suggest spatial working memory and sustained attention are unaffected by the absence of these commissural fibers alone.

## Graphical abstract

The cognitive deficits associated with agenesis of the corpus callosum have been understudied in the BTBR mouse. We studied working memory and sustained attention in this mouse model of AgCC utilizing operant paradigms. Surprisingly, our results from mice with histologically confirmed absence of the corpus callosum and hippocampal commissure were similar to the results from B6 controls.

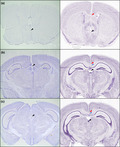

## 1 INTRODUCTION

In a rare congenital disorder known as agenesis of the corpus callosum (AgCC), the axonal fibers that form the brain's largest commissure are either completely absent or fail to fully form, resulting in impaired integration of the cerebral hemispheres (Paul et al., 2007). Callosal projections are predominantly interhemispheric homotopic connections (Aboitiz et al., 1992; McCulloch & Garol, 1941), though a normally developed corpus callosum also facilitates some heterotopic connections and aids in efficient intrahemispheric processing as well (Clarke & Zaidel, 1994; Rakic & Yakovlev, 1968). When the corpus callosum is either completely (cAgCC) or partially (pAgCC) absent, integration of information between the cerebral hemispheres is likely dependent upon the much smaller anterior, posterior, and hippocampal commissures (Bloom & Hynd, 2005; Siffredi et al., 2019).

Agenesis of the corpus callosum affects one in every 4,000 live births (Glass et al., 2008) and is believed to be present in 3%–5% of all neurodevelopmental disorders (Bodensteiner et al., 1994; Jeret et al., 1985). However, the behavioral symptoms of AgCC are highly variable making diagnosis difficult. AgCC can be diagnosed through magnetic resonance imaging (MRI), prenatally through high‐resolution ultrasound, and through other high‐resolution imaging techniques (Santo et al., 2012). AgCC can present with symptoms similar to autism spectrum disorder, and a diagnosis of AgCC does lead to a higher risk for ASD diagnosis (Paul et al., 2014). Overall, AgCC symptoms consistently include deficits in higher‐order cognitive functions, such as learning verbal and visual information, spontaneous memory retrieval, attention processes, as well as social behaviors involving processing and expression of emotions and reciprocal social communication (Brown & Paul, 2019; Paul et al., 2007; Siffredi et al., 2019). Alternative variables, including age and gender, have been observed to be associated with cognitive outcomes in individuals with AgCC (Brown, Panos, & Paul, 2020). Interestingly, when AgCC occurs without other neurological abnormalities, overall cognitive functioning appears to remain largely intact, likely due to interhemispheric integration via extracallosal commissures (Brown & Paul, 2000; Chiarello, 1980; Paul et al., 2007). Brain activation patterns may also be different in individuals with AgCC during cognitive tasks, despite similar performance to controls (Siffredi et al., 2017). Nevertheless, there are notable differences between outcomes on various cognitive tasks, and based upon cumulative evidence, AgCC is thought to cause a generalized deficit in complex behavior and novel problem‐solving while simple behaviors remain intact (Brown & Paul, 2019). For example, impairments in problem‐solving have been observed to increase with the complexity of the problems (Brown & Paul, 2000). Our goal, therefore, was to examine executive functioning in mice with AgCC with regard to increasing complexity. Spatial working memory and sustained attention abilities were compared between BTBR T^+^Itpr3^tf^/J (BTBR) and C57BL/6J (B6) mice using delayed matching‐to‐position (DMTP) and sustained attention operant tasks.

Working memory has a role in processing, encoding, and retrieving information (Baddeley & Hitch, 1974); on the other hand, sustained attention, or vigilance, is the capacity of maintaining a persistent response during continuous and repetitive activity for a period of time (Sohlberg & Mateer, 2001). According to structural and functional neuroimaging findings, prefrontal cortex irregularity may contribute to deficiency in attention regulation and working memory (Bechara et al., 1998; Bush, 2010; Goldman‐Rakic & Friedman, 1991). The dorsolateral prefrontal cortex (DLPEF) in particular plays a major role in executive functions (Durston et al., 2003), including executive control of working memory and sustained attention. Secondary to prefrontal involvement though, other brain regions are recruited to perform specific aspects of both working memory and attention. For example, neuropsychological evidence has associated the left inferior frontal gyrus and insula with rehearsal (Ardila, 1992; Benson, 1979; Damasio, 1981; Dronkers, 1996); and the ventral portion of the inferior parietal cortex with storage (Damasio & Damasio, 1983; Green & Howes, 1977; Sakurai et al., 1998) during verbal working memory. Thakral and Slotnick (Thakral & Slotnick, 2009) also found that the parietal cortex, especially the superior parietal lobule and inferior parietal lobule, is related to visual attention in both shifting attention and sustained attention.

DMTP tasks are well‐accepted paradigms that assess spatial working memory performance. By using an operant procedure in which animals have to remember which of two retractable levers have most recently been presented, nonspecific deficits in performance (i.e. motor impairments) can be separated from mnemonic impairments through analysis of forgetting curves that are associated with increasing delay interval (Sahgal, 1987b). As trials progress and the memory load increases, animals need to store more information in their working memory, and as a consequence, accuracy decreases. The operant DMTP task has certain advantages over maze tasks, including a higher number of trials per session, as well as the ability to fully automate the task and thus assess performance with high accuracy (Estape & Steckler, 2001).

Initial measures created to test vigilance in animals failed to accurately measure subjects’ abilities to discriminate between signal and nonsignal events (McGaughy & Sarter, 1995). McGaughy and Sarter were able to create an operant task that accurately measured vigilance in rats, based off of criteria used for well‐established human paradigms measuring sustained attention (Parasuraman et al., 1989). McGaughy and Sarter's task was later adapted as a valid paradigm for measuring sustained attention abilities in mice (Martin et al., 2006). In this task, the mice are required to identify and distinguish between signal and nonsignal stimuli. Lights are presented as “signals” for different durations of time, and the mice are trained to lever press based upon signal and nonsignal events. Differing lengths of stimulus duration indicate sustained attention abilities, with responses being classified as hits, correct rejections, misses, or false alarms. Performance is a function of signal length, with shorter stimulus durations resulting in a higher number of misses (McGaughy & Sarter, 1995; Sarter et al., 2001).

We chose to compare the BTBR T + tf/J and the C57BL/6J inbred mouse strains. The complete absence of the corpus callosum in BTBR mice makes this one of the best available mouse strains to study AgCC. Several other mouse strains reported to exhibit AgCC have variable penetrance of the morphological abnormalities (Wahlsten et al., 2003). Beyond its utility as a model for AgCC, the BTBR mouse strain has also been used as a model to study Autism Spectrum Disorder (ASD). Several studies have explored the relationship between ASD and the corpus callosum (CC), with some studies reporting a significant reduction in the size of CC in patients with ASD (Chung et al., 2004; Vidal et al., 2006; Waiter et al., 2005) and others not finding any significant relationship (Elia et al., 2000; Herbert et al., 2004; Paul et al., 2014). Moy et al. (2007) was the first to utilize this strain as a model in ASD research and since this time numerous studies have confirmed reduced social behaviors in this strain among other behavioral differences (e.g., McFarlane et al., 2008; Pearson et al., 2011; Scattoni et al., 2008, 2011; Silverman et al., 2010). However, social behavior deficits may be part of an overall deficit in motivated behavior (Martin et al., 2014; McTighe et al., 2013). The B6 inbred mouse strain was chosen as a control strain because it has normal commissural fibers and has been commonly used as a control strain for comparisons with BTBR mice.

Based on previous research, we hypothesized that deficits in executive functions would emerge as task difficulty increased within testing paradigms. Specifically, we predicted that as complexity increased through a longer delay interval in the DMTP task and through a shorter signal duration in the sustained attention task, differences in accuracy would emerge between the two strains on both tasks, with the BTBR mice performing worse than the B6 mice due to AgCC.

## 2 MATERIALS AND METHODS

### 2.1 Test subjects

A total of 54 mice were included in the study from the BTBR T^+^Itpr3^tf^/J (IMSR Cat# JAX:002282, RRID:IMSR_JAX:002282) and C57BL/6J (IMSR Cat# JAX:000664, RRID:IMSR_JAX:000664) inbred strains. Separate mice were used in each task with 22 BTBR and 12 B6 mice assigned to the DMTP task and 12 BTBR and eight B6 mice assigned to the sustained attention task. After mice reached at least 8 weeks of age, they were food deprived to between 80% and 85% of their baseline body weight, habituated to the apparatus, and then trained to perform the tasks. In an effort to reduce animal usage, only female mice were tested in the DMTP Task and only male mice were tested in the sustained attention task. Additionally, a smaller number of mice were tested in the sustained attention task than the DMTP task because similar results were observed between BTBR and B6 mice after the first groups of mice completed testing and further testing was therefore not justified. While genotype by sex interactions was not controlled for in this study, previous research found that both male and female mice perform similarly on the DMTP and sustained attention tasks (Martin et al., 2004, 2006). These mice were obtained from the principal investigator's own breeding colonies which were originally established from breeder pairs obtained from Jackson Laboratories (Bar Harbor, Maine). All mice were housed in a vivarium with a set 14:10 hr light:dark cycle in a climate‐controlled setting with temperature maintained at 20°C. All testing was conducted during the light phase of the cycle. All mice were housed in groups of 2–4, but were individually housed during behavioral testing due to food deprivation procedures. Mice were housed in ventilated cages (OptiMICE; Animal Care Systems) with Sani‐Chips bedding (PJ Murphy) and full Ancare 6002 nestlets (Ancare). They were given a restricted diet of pellet feed (Purina 5001; Cargill) and water ad libitum. Additionally, mice were identified via ear tags for the DMTP task and via tail tattoos for the sustained attention task. All mice were treated in accordance with the NIH guidelines for the care and use of animals in research, and all procedures were approved by the Azusa Pacific University Institutional Animal Care and Use Committee.

### 2.2 Testing apparatus

All behavioral testing was conducted using four identical operant chambers (Model ENV‐307; 15.9 cm long, 14 cm wide, 12.7 cm tall; Med Associates; St. Albans, VT). Each was equipped with two retractable levers (Model E23‐07) positioned to the left and right of the food magazine such that when extended, each lever was 1.0 cm from the wall, and 2.5 cm above the grid floor. A liquid dipper presented reinforcement consisting of 0.02 ml of evaporated milk sweetened with 0.2% sucrose solution into a food magazine centered on the short wall and adjacent to each lever. The duration of dipper presentation was 7 s throughout training and testing. A white light located within the food receptacle signaled reinforcement delivery. The chambers were equipped with a houselight, located on the rear wall opposite of the food magazine and lever, which served as a stimulus to signal when the experimental contingencies were in effect and when an incorrect response was made (5‐s time‐out signaled by the houselight turning off). Each experimental chamber was enclosed within a sound‐attenuating melamine cubicle with a small exhaust fan to provide continuous airflow and background noise. Data from the DMTP and Sustained Attention Operant Tasks were collected using the Med PC system (Med Associates; St. Albans, VT) which controlled the delivery of the liquid reinforcer and recorded lever pressing and entries into the food magazine.

### 2.3 Operant testing procedures

The operant testing procedures are described below and a summary of the procedures is shown in Table 1.

**TABLE 1:** Breakdown of operant procedures and criterion for advancement

Procedure	Step 1	Step 2	Step 3	Step 4	Criterion
Lever‐Press Training 30‐min sessions	Both levers extend into test chamber.	Mice are manually shaped to press either lever.	Successive approximations of desired goal are rewarded by presentation of food reinforcement.	Mouse nosepokes into food magazine and receives reward.	10 lever presses on either lever over three consecutive sessions.
DMTP Zero‐Delay Stage 40‐min sessions	One lever extends into test chamber.	Mouse presses lever and then nosepokes into illuminated food magazine.	No food reinforcement is presented but both levers now extend.	Mouse presses the original lever and food reinforcement is delivered. Mouse presses opposite lever and houselight turns off for 5 s.	Greater than 90% correct trials over 3 consecutive days.
DMTP Delayed Matching Stage 40‐min sessions	One lever extends into test chamber.	Mouse presses lever and then nosepokes into illuminated food magazine.	No food reinforcement is presented but both levers now extend after a variable delay. Two different delay sets are utilized in separate rounds of testing.	Mouse presses the original lever and food reinforcement is delivered. Mouse presses opposite lever and houselight turns off for 5 s.	Data analyzed from final 10 testing sessions after asymptotic performance is reached.
Sustained Attention Training 144 trials per session with 72 signal events and 72 nonsignal events	A central stimulus light illuminates for 500 ms representing a signal event or remains off representing a nonsignal event.	Following a 2 s delay, both levers extend into the test chamber until a lever press occurs or 4 s elapses.	Mice are rewarded with food reinforcement by pressing the left lever after a signal event or the right lever after a nonsignal event.	Correct responses: Signal = hits Nonsignal = correct rejections Incorrect responses: Signal = misses Nonsignal events = false alarms	Correct response on greater than 59% nonsignal and 500 ms trials in 3 of 5 consecutive sessions.
Sutained Attention Testing 144 trials per session with 72 signal events (18 per duration) and 72 nonsignal events	A central stimulus light illuminates for 50, 75, 100, or 500 ms representing a signal event or remains off representing a nonsignal event.	Following a 2 s delay, both levers extend into the test chamber until a lever press occurs or 4 s elapses.	Mice are rewarded with food reinforcement by pressing the left lever after a signal event or the right lever after a nonsignal event.	Responses are recorded as described in training but signal events are categorized by duration.	Data analyzed from 3 of 5 consecutive sessions with correct response on greater than 59% nonsignal and 500 ms trials.

#### 2.3.1 Lever‐press training procedure

In 30‐min lever‐press training sessions, mice were manually shaped to press one of the two reinforcement levers to receive the liquid reinforcement by rewarding successive approximations of the desired response. Following successful completion of lever‐press training, defined as at least 10 lever presses for three consecutive days, mice were trained in either the DMTP or sustained attention operant tasks.

#### 2.3.2 DMTP procedure

Mice were trained to perform the DMTP task in two stages: zero‐delay training and delayed matching (Martin et al., 2004). In the zero‐delay training stage, mice were first presented with one of two levers (the sample lever) on the left or right of the food magazine. Upon a lever press, the food magazine illuminated (reinforcement was not delivered) and the mouse was required to nose‐poke into the food magazine in order to initiate the choice phase. In this choice phase, both levers were extended and a correct response (matching the original sample lever) resulted in magazine light illumination and delivery of the liquid reward. An incorrect response (choosing the nonmatching lever) resulted in a 5‐s time‐out (extinguishing the houselight) without the liquid reward. An intertrial interval of 5 s was used in the 40‐min session. Training continued until mice achieved >90% correct trials on three consecutive days.

Upon advancing from the zero‐delay training stage, mice began the delayed matching stage of the experiment. This stage was very similar to stage one with the exception that time delays were introduced between the sample (first lever press) and choice (second lever press) phases. All mice were tested daily with each delay set until they reached asymptotic levels of performance and then continued to be tested daily for 10 consecutive days. The following intermittent delay sets were utilized: set 1 = 24 *[0,2,4,8,12,18,24]* seconds and set 2 = 36 *[0,6,12,18,24,30,36]* seconds. These delay sets were run in progression from set 1 to 2. Following the completion of each daily training or testing session, all mice were returned to their home cage and received sufficient food to maintain their food‐deprived weights (approximately 3 g of Purina 5001 mouse chow).

#### 2.3.3 Sustained attention procedure

The sustained attention operant task was also carried out in two stages: sustained attention training and sustained attention testing (Martin et al., 2006). First, the mice had to distinguish a signal event consisting of a stimulus light that illuminated for 500 ms from a nonsignal event in which there was no illumination. Two seconds following each signal or nonsignal event, two response levers were extended into the chamber. Mice were then reinforced following a signal event by pressing the left response lever (recorded as a hit) and following a nonsignal event by pressing the right response lever (recorded as a correct rejection). An incorrect response to a signal event was recorded as a miss, and an incorrect response to a nonsignal event was recorded as a false alarm. If no lever press was made, the levers retracted after 4 s. Each session involved a total of 144 trials that were pseudorandomly presented to ensure an equal number of signal and nonsignal trials. After mice correctly responded to >59% of both the nonsignal events and the 500 ms signal events for at least three of five consecutive sessions, they moved into sustained attention testing.

The sustained attention testing procedure was very similar to the training stage with the exception that additional signal event durations were introduced. Instead of having only the 500 ms signal event, the signal events were pseudorandomly presented in four different durations: 50, 75, 100, and 500 ms. Each signal duration was presented 18 times in a session so that there were 72 signal and 72 nonsignal events that occurred in a pseudorandom sequence. Testing was complete once mice correctly responded to >59% of all of the nonsignal events but only the 500 ms signal events for at least three of five consecutive sessions.

### 2.4 Histology

Following the completion of DMTP testing, a subgroup of 16 mice were chosen for histological analysis, eight of which successfully completed testing, and eight that failed to advance to the testing stage. Mice were overdosed with anesthesia (Avertin) and transcardially perfused with phosphate buffered saline followed by a 4% Paraformaldehyde fixative. When the subjects were properly fixed, the heads were removed using surgical scissors and the skulls were cut midsagittally along the dorsal side of the cranium and the brains were removed. Some of the brain samples were then placed in glucose solutions of increasing concentrations and then flash frozen with OCT compound and liquid nitrogen and coronally sectioned with a cryostat before being stained with cresyl violet for histological analysis. However, an alternate histological technique was employed on most of the BTBR brain samples to improve tissue integrity for photographic purposes. These samples were cleared with a series of xylenes and infiltrated with paraffin, followed by embedding in paraffin blocks. Paraffin embedded brains were sectioned in the coronal plane using a microtome set for 8 µm thickness. Sections were then mounted on Superfrost++ slides (Fisher Scientific), cleared with xylenes, hydrated through descending alcohol concentrations, and then stained with cresyl violet. After rinsing with dH_2_0, the sections were dehydrated through ascending alcohol concentrations before being cleared with xylenes. Glass coverslips were then applied with Permount.

### 2.5 Research design and statistical analysis

#### 2.5.1 Lever‐press training procedure

The number of days to reach criterion to advance from the lever‐press training procedure was compared using independent samples *t* tests. Equality of variance was assessed using Levene's test.

#### 2.5.2 DMTP task

Data from the last 10 days of testing after each mouse reached asymptotic performance in each delay set were used for statistical analyses. The mean percentage of correct responses at each delay interval across these 10 trials were used to determine delay dependent effects on spatial working memory. Mixed‐model ANOVAs with pairwise comparisons and Bonferroni corrections using IBM SPSS statistical software were used to evaluate group differences in performance. In each model, genotype was the between‐subjects factor and delay interval was the within‐subjects factor. In addition, the linear relationship between the percentage of correct responses and delay interval was demonstrated using Pearson's *R* for each genotype and each delay set.

We also calculated "index Y" at each delay for the 24 s delay set (Estape & Steckler, 2001; Sahgal, 1987a, 1987b) defined as the sum of the percentage of left correct responses minus the percentage of right correct responses divided by the sum of the percentage of left correct responses plus the percentage of right correct responses. The range of index *Y* is +1 to −1, and scores around zero indicate lower bias and better stimulus control. A negative index *Y* represents a right response bias while a positive index *Y* represents a left response bias. Thus, index *Y* could be used to determine whether delay‐induced declines in performance were due to either a greater demand on working memory or a bias for one lever over the other.

#### 2.5.3 Sustained attention task

Only data from the three sessions of the sustained attention testing stage in which mice correctly responded to >59% of both the nonsignal events and the 500 ms signal events were utilized for statistical analyses. The data were analyzed by first calculating a measure of accuracy of task performance in the relative number of hits [hits/(hits + misses)] and relative number of correct rejections [correct rejections/(correct rejections + false alarms)] for each test session at all signal events. Comparisons were then made using independent samples *t* tests and repeated‐measures ANOVA with pairwise comparisons and Bonferroni corrections using IBM SPSS statistical software. As the sustained attention and DMTP tasks were conducted separately using a different set of mice, the Bonferroni corrections were independently applied to the results of each task.

## 3 RESULTS

### 3.1 Lever‐press training

Out of the 34 BTBR mice that were trained in the lever‐press training procedure for both studies, there were four BTBR mice (three assigned to DMTP and one assigned to Sustained Attention) that did not learn to lever press for a food reward after 60 days of training and were thus removed from further testing. In contrast, all 20 B6 mice learned to lever press for a food reward. For those mice that did successfully advance from this procedure, the number of daily sessions required to reach criterion of 10 lever presses over three consecutive days was significantly higher for the 30 BTBR (*M* = 32.4, *SD* = 17.2) versus the 20 B6 (*M* = 12.0, *SD* = 7.2) mice (*t* = 5.787, *df* = 41.925, *p* < .001; equality of variance not assumed). Out of these 30 BTBR mice, 19 were assigned to the zero‐delay training stage of the DMTP task, but only 14 successfully reached criterion to advance to the testing stage after approximately three months of testing. In contrast, all 12 B6 mice assigned to the DMTP task advanced to the testing stage. Of the 11 BTBR mice that were assigned to sustained attention training, only eight successfully reached criterion to advance to the testing stage. In comparison, six out of 8 B6 mice advanced to the testing stage.

### 3.2 DMTP

**FIGURE 1:**
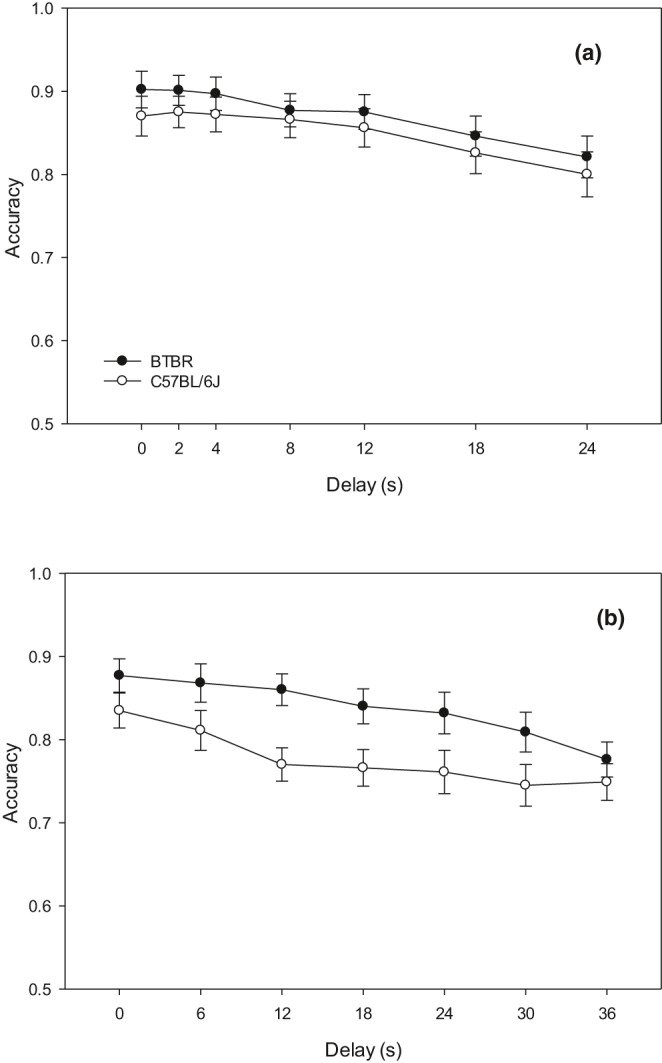
Line graphs comparing performance on the DMTP paradigm of the BTBR and B6 mice. (a) In the 24‐s delay set, there was a significant effect of delay duration on accuracy for all mice (*F* = 23.375, *df* = 6,144, *p* < .001). However, there was no interaction between delay duration and mouse strain (*F* = 0.281, *df* = 6,144, *p* = .945). (b) In the 36 s delay set, ANOVA revealed there was also a significant effect of delay duration on accuracy (*F* = 27.136 *df* = 6,138, *p* < .001). There was also an interaction between delay duration and mouse strain (*F* = 2.744, *df* = 6,138, *p* = .015). However, pairwise comparisons with Bonferroni corrections did not reveal any significant differences between mouse strains at each delay duration

Results from the 24‐s delay set showed that there was a significant effect of delay duration on accuracy for all mice (*F* = 23.375, *df* = 6,144, *p* < .001; Figure 1a). However, there was no interaction between the delay duration and mouse genotype (*F* = 0.281, *df* = 6,144, *p* = .945). Calculations of index *Y* at each delay in the 24 s delay set revealed no lever response bias in either mouse strain (Table 2).

**TABLE 2:** Index *Y* at each delay in the 24 s delay set indicated no lever response bias in either the BTBR or B6 strain

	0 s	2 s	4 s	8 s	12 s	18 s	24 s
BTBR	0.0077	−0.0015	0.0193	0.0079	0.0062	0.0210	0.0043
B6	−0.0147	−0.0356	−0.0406	−0.0274	−0.0461	−0.0218	−0.02842

For the 36‐s delay set of the DMTP task, only 13 BTBR mice and 12 B6 mice completed testing as 1 BTBR mouse was removed from testing due to health concerns. The results from the 36 s delay set also revealed a significant effect of delay duration on accuracy (*F* = 27.136 *df* = 6,138, *p* < .001; Figure 1b). For this delay set, there was also an interaction effect between delay duration and mouse genotype (*F* = 2.744, *df* = 6,138, *p* = .015). However, pairwise comparisons with Bonferroni corrections did not reveal any significant differences between the mouse strains at each delay duration.

**FIGURE 2:**
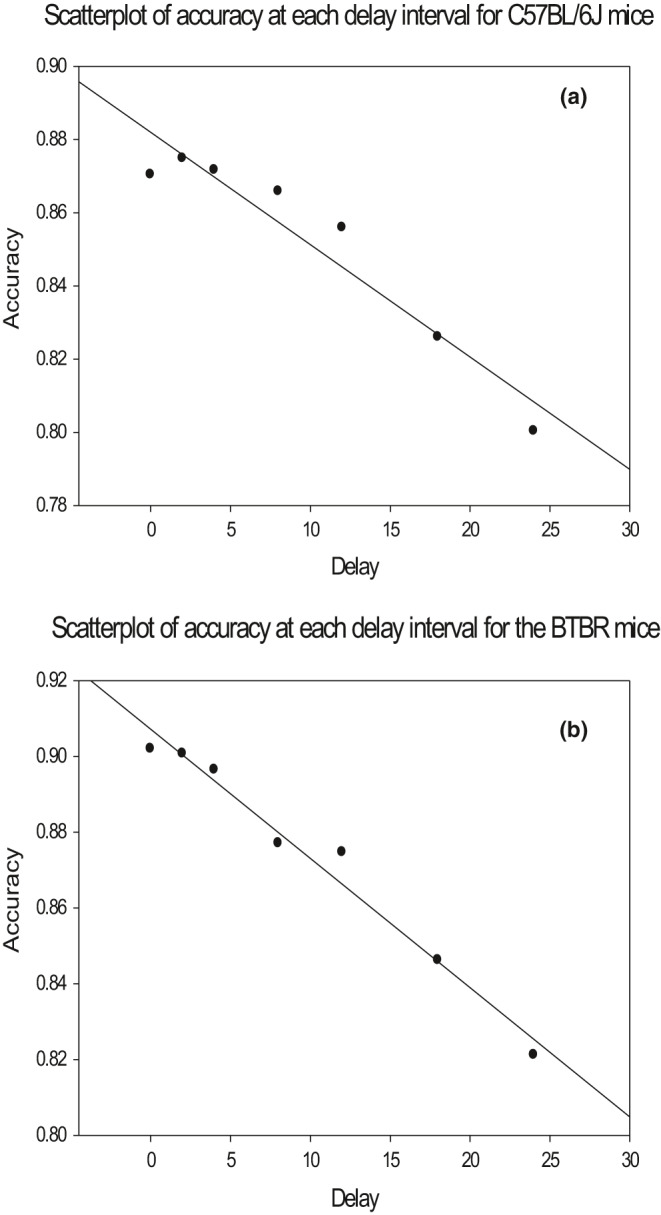
Scatterplots demonstrating the relationship between delay interval and accuracy for both mouse strains in the 24‐s delay set. Pearson's correlations demonstrated a highly significant negative relationship between delay interval and accuracy for both (a) C57BL/6J (*r* = −.959, *p* = .001) and (b) BTBR (*r* = −.988, *p* < .001) mice. These relationships were also highly significant for the 36‐s delay set (data not shown; B6: *r* = −.918, *p* = .004; BTBR *r* = −.972, *p* = .001)

For the B6 mouse strain, Pearson's correlations demonstrated a highly significant negative relationship between delay interval and accuracy on both the 24 s (Figure 2a) and the 36 s delay sets (24 s delay set: B6 *r* = −.959, *p* = .001; 36 s delay set: B6: *r* = −.918, *p* = .004). Similarly, accuracy at each delay interval in the BTBR mice also resulted in a highly significant negative relationship between the delay interval and accuracy. Pearson's correlations demonstrate this relationship on both the 24‐s (Figure 2b) and 36‐s delay sets (24 s delay set: BTBR: *r* = −.988, *p* < .001; 36 s delay set: BTBR: *r* = −.972, *p* = .001).

### 3.3 Sustained attention

The results of the 8 BTBR and 6 B6 mice that completed the sustained attention task are shown in Table 3. These results were used to calculate the mean accuracy of task performance which was then compared through independent samples *t* tests. There were no significant differences between BTBR and B6 strains in the performance of sustained attention across all levels of the stimulus. Mean accuracy was similar between the strains for 50 ms (*t* = −0.696, *df* = 9.539, *p* = .503), 75 ms (*t* = −0.549, *df* = 12, *p* = .593), 100 ms (*t* = −0.555, *df* = 12, *p* = .589), 500 ms (*t* = −0.585, *df* = 12, *p* = .569), and nonsignal (*t* = −0.208, *df* = 12, *p* = .839) trials of the sustained attention test.

**TABLE 3:** The relative percentage of hits (hits/hits + misses) and relative percentage of correct rejections (correct rejections/correct rejections + false alarms) for the BTBR and B6 mice

	Relative hits 50 ms	Relative hits 75 ms	Relative hits 100 ms	Relative hits 500 ms	Relative correct rejections
BTBR	41.6%	48.0%	49.4%	75.5%	70.8%
B6	45.6%	51.7%	53.2%	71.5%	72.0%

**FIGURE 3:**
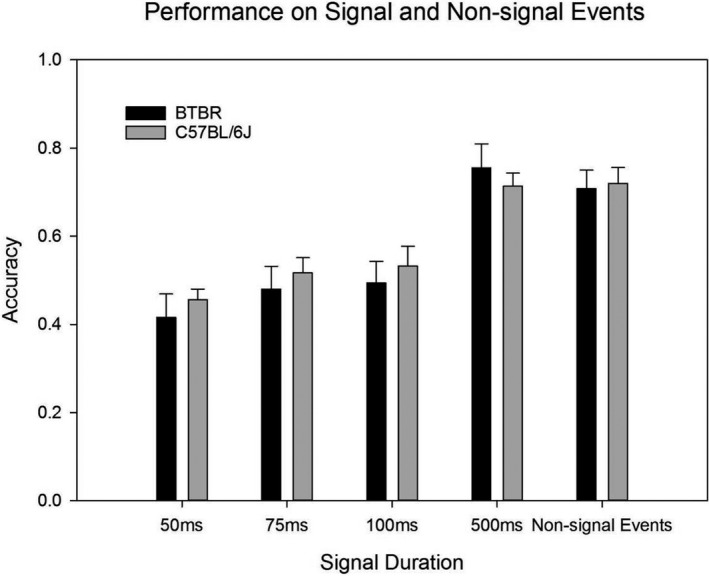
Bar graphs comparing performance on the Sustained Attention paradigm of the BTBR and B6 mice. Independent samples *t* tests showed no significant differences between BTBR and B6 strains in relative hits or relative correct rejections across all levels of the stimulus. There was a significant effect of signal duration on performance accuracy observed for both strains (BTBR: *F* = 51.402, *df* = 3,21, *p* < .001; B6: *F* = 12.501, *df* = 3,15, *p* < .001)

The within‐subject mean accuracies of task performance at each level were compared through repeated‐measures ANOVA. There was a significant effect of signal duration on performance accuracy observed for both strains (BTBR: *F* = 51.402, *df* = 3,21, *p* < .001; B6: *F* = 12.501, *df* = 3,15, *p* < .001). However, for the BTBR strain, pairwise comparison with Bonferroni corrections revealed that only the 50 ms trials had significant differences with other signal durations. For the B6 strain, pairwise comparison with Bonferroni corrections showed that the only significant difference was observed between the 50 and 500 ms trials (Figure 3).

### 3.4 Histological analysis

**FIGURE 4:**
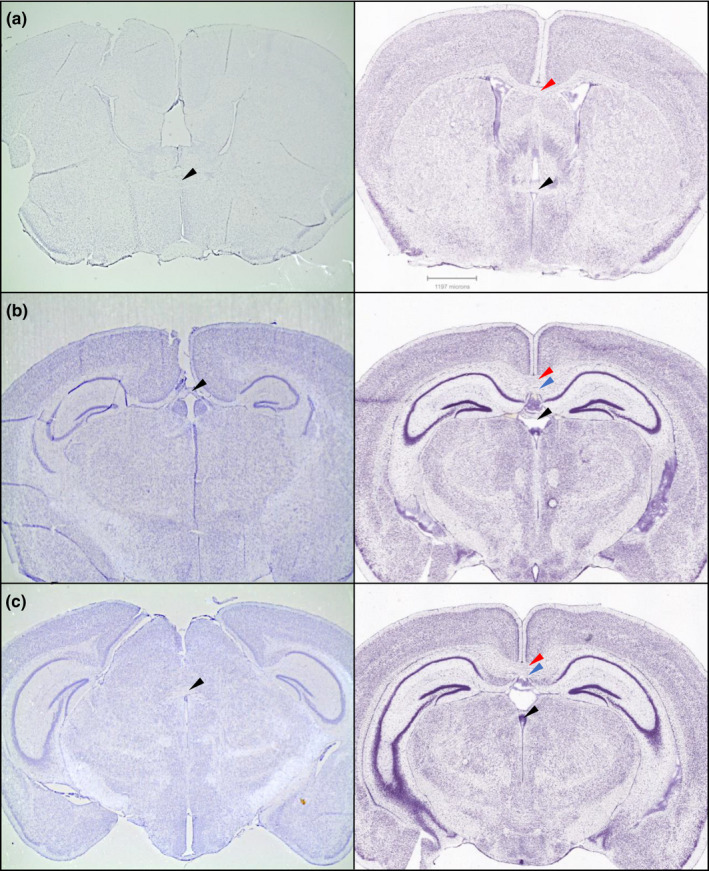
Representative coronal brain sections from BTBR mice (Left) that depict the absence of the corpus callosum and hippocampal commissure but the presence of other commissural fibers. Brain sections were stained with cresyl violet and are arranged from anterior to posterior sections at approximately Bregma 0.2 mm, Bregma −2.0 mm, Bregma −2.2 mm (a–c). Comparison sections #55, 77, & 79 from B6 mice (Right) were obtained from the Allen Mouse Brain Atlas © 2004 Allen Institute for Brain Science. Allen Mouse Brain Atlas. Available from: http://atlas.brain‐map.org/. The arrowheads point to commissural fibers: (a) red = corpus callosum, black = anterior commissure; (b) red = corpus callosum, blue = hippocampal commissure, black = habenular commissure, and (c) red = corpus callosum, blue = hippocampal commissure, black = posterior commissure. The commissural fibers of the corpus callosum and hippocampal commissure are clearly absent in the BTBR brain sections and the hippocampus is laterally displaced. The scale bar is from the Allen Mouse Brain Atlas and is approximate for the BTBR brain sections

Upon conclusion of DMTP testing, a sampling of 16 BTBR mice, eight that completed testing and eight that failed to advance to the testing stage, were selected for perfusion and sectioning to examine brain tissue. Histological analyses showed that all BTBR mice that were examined lacked the commissural fibers of the corpus callosum and the hippocampal commissure. Anterior, habenular, and posterior commissural fibers remained intact (see Figure 4).

## 4 DISCUSSION

Agenesis of the corpus callosum has been thought to be linked with a generalized deficit in complex behaviors. In this study, we aimed to examine this within the C57BL/6J and BTBR T + tf/J mouse strains through performance on two operant paradigms of executive functioning. We predicted that as complexity increased through a longer delay interval in the DMTP task and through a shorter signal duration in the sustained attention task, differences in accuracy would emerge between the two strains on both tasks, with the BTBR mice performing worse than the B6 mice due to AgCC.

The results from these studies did not support our hypotheses. As expected, both the B6 and BTBR strains demonstrated a predictable decline in performance on the DMTP task as the delay interval increased. However, there was no significant difference in the performance measures between the two strains, even at the maximum delay intervals. Likewise, the results of the sustained attention task did not yield significant differences in performance between the B6 and BTBR strains. Both strains demonstrated a decrease in accuracy as the duration of the stimulus light shortened; however, both strains performed similarly in all signal and nonsignal tasks, even at the 50 ms minimum signal duration.

Taken together, these results provide evidence that for mice, the executive functions of spatial working memory and sustained attention are not significantly impacted by absence of the corpus callosum alone. Other studies comparing the BTBR and B6 mouse strains have found significant differences in cognitive abilities including learning (Amodeo et al., 2012), memory (MacPherson et al., 2008; Ribeiro et al., 2013; Steinmetz et al., 2018), and attention (Chao et al., 2018; McTighe et al., 2013). Our results were surprising, especially when compared to the previously reported deficits in working memory during handedness learning (Ribeiro et al., 2013) and visuospatial attention in the five‐choice serial‐reaction time task (McTighe et al., 2013). And yet, our operant tasks involved higher demands on working memory and vigilance than these other tasks (e.g. to‐be‐remembered stimuli durations of up to 36 s in the DMTP task and signal durations ranging from 50 to 500 ms in the sustained attention task compared to a range of 400 to 4,000 ms in the five‐choice serial‐reaction time paradigm utilized by McTighe et al.).

While we were surprised at how well the BTBR mice performed on the DMTP and sustained attention tasks, several of the mice initially selected for this task failed to reach criterion to advance to the testing stages. Specifically, 8 out of 22 BTBR mice on the DMTP task and four out of 12 BTBR mice on the sustained attention task failed to advance to the testing stage, with a total of four BTBR mice failing to advance past lever‐press training. Of those mice that achieved lever‐press training criterion, the BTBR mice required significantly more training sessions than the B6 mice. This finding is consistent with our previous research on the BTBR strain using an operant task (Martin et al., 2014), as well as that of others demonstrating increased population variance within this strain (McTighe et al., 2013; Rutz & Rothblat, 2012). This has also been observed in humans with AgCC, with variability in presentation of general intellectual and executive functioning abilities (Siffredi et al., 2018). A key distinction between our work and others is that we removed the BTBR mice from our study that did not reach criterion after a preset number of training days, reducing the population variance in our testing data. We acknowledge that within the AgCC population there may be greater variability in cognitive functioning, as our testing sample reflected only those mice with the cognitive capability to learn to lever press. Given that this specific subset of mice advanced to task completion, our results suggest that at least some of the cognitive impairments previously reported for the BTBR strain are driven by a different subset of mice within this inbred population.

We hypothesized that the previously reported variable size of the hippocampal commissures in the BTBR strain may relate to the variation in associative learning observed in these mice (Wahlsten et al., 2003). To test this hypothesis, we chose to histologically explore a subset of mice from the DMTP task. Histological investigation of the BTBR mouse brain demonstrated the complete absence of the corpus callosum and hippocampal commissures in all BTBR mice that were examined, regardless of associative learning capability. These findings suggest the absence of the hippocampal commissure is not likely linked to the variability in cognitive ability observed within this strain, and thus, the neuropathology underlying this variability remains unknown.

The results of the DMTP and sustained attention tasks indicate that while the prefrontal cortex is well known for its role in executive functions, and is heavily interhemispherically connected via the corpus callosum, it does not seem to depend upon these connections for normal functioning of at least these two executive functions. Moreover, several studies on human participants demonstrated an unclear pattern of the relationships between the corpus callosum and working memory and sustained attention. Studies on sustained attention suggested an atypical corpus callosum may result in sustained attention deficits, but the results are inconsistent (Ellenberg & Sperry, 1979; Semrud‐Clikeman et al., 1994). Other studies indicate conflicting findings on the impact of AgCC on working memory (Labadi & Beke, 2017; Paul et al., 2016; Siffredi et al., 2017). To further understand the role of AgCC within executive functioning, future research should focus on other executive functions, such as the organization of goal‐directed action, impulse control, cognitive flexibility, reasoning, and problem‐solving. Indeed, there is some evidence indicating deficits in cognitive flexibility in the BTBR strain (Karvat & Kimchi, 2012; Rutz & Rothblat, 2012).

## 5 CONCLUSION

Overall, these studies explored the impact of agenesis of the corpus callosum on spatial working memory and sustained attention, two aspects of executive functioning. The delayed matching‐to‐position task is a well‐established paradigm for assessing spatial working memory in both animal and human research, and the use of signal and nonsignal events in an operant task is an established model for examining sustained attention. The results provide evidence that spatial working memory and sustained attention are not impacted by absence of the corpus callosum and hippocampal commissures alone. Although this is counter to what was originally predicted, the findings in this study point to the need for further research to more clearly specify executive functioning deficits associated with AgCC.

## CONFLICT OF INTEREST

The authors report no conflict of interest.

## AUTHOR CONTRIBUTION

LAM designed the research experiments and involved in data acquisition, data analysis, and manuscript development. F‐WH assisted with data acquisition, data interpretation, and manuscript development. BH assisted with the data interpretation and manuscript development. MG and HS assisted with data acquisition and manuscript development. JK assisted with the research design and manuscript development.

### Peer Review

The peer review history for this article is available at https://publons.com/publon/10.1002/brb3.1933.

## Data Availability

The data that support the findings of this study are available from the corresponding author upon reasonable request.
